# Two vicious circles contributing to a diagnostic delay for tuberculosis patients in Arkhangelsk

**DOI:** 10.3402/ehtj.v7.24909

**Published:** 2014-08-26

**Authors:** Vladimir N. Kuznetsov, Andrej M. Grjibovski, Andrej O. Mariandyshev, Eva Johansson, Gunnar A. Bjune

**Affiliations:** 1 Institute of Health and Society, University of Oslo, Oslo, Norway; 2 Department of International Public Health, Norwegian Institute of Public Health, Oslo, Norway; 3 Department of Tuberculosis, Northern State Medical University, Arkhangelsk, Russia; 4 Karolinska Institute, Stockholm, Sweden

**Keywords:** tuberculosis, patient delay, health system delay, grounded theory

## Abstract

**Setting:**

Delay in tuberculosis (TB) diagnosis increases the infectious pool in the community and the risk of development of resistance of mycobacteria, which results in an increased number of deaths.

**Objective:**

To describe patients’ and doctors’ perceptions of diagnostic delay in TB patients in the Arkhangelsk region and to develop a substantive model to better understand the mechanisms of how these delays are linked to each other.

**Design:**

A grounded theory approach was used to study the phenomenon of diagnostic delay. Patients with TB diagnostic delay and doctors–phthisiatricians were interviewed.

**Results:**

A model named ‘sickness trajectory in health-seeking behaviour among tuberculosis patients’ was developed and included two core categories describing two vicious circles of diagnostic delay in patients with TB: ‘limited awareness of the importance to contact the health system’ and ‘limited resources of the health system’ and the categories: ‘factors influencing health-seeking behaviour’ and ‘factors influencing the health system effectiveness’. Men were more likely to report patient delay, while women were more likely to report health system delay.

**Conclusions:**

To involve people in early medical examinations, it is necessary to increase alertness on TB among patients and to improve health systems in the districts.

Diagnostic delay is defined as the duration from the first appearance of symptoms to the initiation of treatment. It is a considerable problem in tuberculosis (TB), as it increases the infectious pool in the community (increases the time a patient stays infected) and also the mortality rate (1). This includes patient delay (PD) (from the onset of symptoms to the first contact with the health services) and health system delay (HSD) (from the first visit to the health services to the initiation of correct treatment) (2, 3).

HSD is often associated with a patient's visit to the health system's primary level of care without receiving a correct diagnosis (4, 5). This is related to not following the national diagnostic algorithm (6), unavailability of modern diagnostic tests (7) and difficulties in recognising TB symptoms in coexistence with chronic cough and/or other lung diseases (4, 5). HSD is also associated with an initial visit to a low-level healthcare facility or a private practitioner (or traditional healer) without receiving a correct diagnosis (4, 5, 8, 9). Stigmatisation is another barrier that plays a negative role in health-seeking behaviour (10). An urban setting, where lack of communication between different professionals is common, complicates the situation (11, 12). The use of antibiotics in the case of smear-negative pulmonary TB also increases the HSD (13).

PD depends on patients’ knowledge, attitudes and beliefs (14), which are associated with rural residence, low access to health centres, old age, poverty, gender (depending on country customs), alcohol and other substance dependence, immigration background, low education, low awareness of TB, self-treatment and stigma (1, 4, 11, 15). In some contexts, TB DD is associated with fear of contagion and of rejection by health professionals (16). Smokers have a higher risk of delay since they are accustomed to coughing and are therefore less concerned (1, 9, 17). According to other research, health education and awareness of symptoms may not increase a patient's willingness to contact the TB care system for populations with complex health and social needs; and complex pathways are necessary to support people seeking diagnosis and treatment (18).

The factors listed above are more common in vulnerable groups of people such as ethnic minorities, immigrants, and alcohol or drug users (11, 12, 19–21). Alcohol and other substance dependence are the most commonly reported behavioural risk factors for TB DD. Patients who abuse alcohol are problematic for identifying at-risk contacts and TB screening. They are reluctant to seek routine medical care, tend to remain contagious longer and have a high level of treatment failure (22). In the Arkhangelsk region, the HSD median time was less than 2 weeks, while PD was 6 weeks in passive case findings in 2005–2011 (23).

In Russia, both the TB incidence and mortality have decreased during the past decade (24), but the indicators still remain higher than in most industrialised countries (25), and drug-resistant forms of *Mycobacterium tuberculosis* are a serious threat (35.5% of new TB cases) (25, 26). Patients with a resistant form of TB seem to be more likely to abuse alcohol (27, 28).

Cough duration for more than 2 weeks (the internationally accepted criteria of TB DD) often does not motivate smokers enough to contact the health system as they consider it a ‘normal’ thing to cough (29, 30). It is common that such patients first get the diagnosis of respiratory tract infection. Pulmonary TB comes up only as a ‘second line’ possibility, when the patient does not respond to treatment of the respiratory tract infection (31, 32).

Although DD has been studied, there is a lack of information about the interconnection between the two types of delay both in Russia and internationally. The purpose of this study was to describe patients’ and district doctors’ perceptions of DD among TB patients in the Arkhangelsk region and to develop a substantive model for better understanding of the mechanisms driving the connection between patient and HSDs.

## Methodology

### Study design

In focus group discussions (FGDs) and in-depth interviews, participants were asked questions about their perceptions of health, how they perceived TB and TB services and what led them to the late diagnosis. A grounded theory (GT) (33) approach was used for data collection and analysis to generate new theory of factors contributing to mechanisms of DD on the path from symptoms onset to correct diagnosis of TB (33, 34). Based on an inductive approach, a model of delay routes among TB patients in Arkhangelsk was developed (33–35).

The research team consisted of a triangulation of researchers with different backgrounds from Russia, Sweden and Norway: two TB specialists (G.B. and A.M.), one nurse (E.J.) with expertise in public health and qualitative research, one epidemiologist (A.G.) and one psychiatrist with public health experience [principal investigator (PI), V.K.].

### Setting

The Arkhangelsk region is situated in the north of Russia, 1,200 km north of Moscow and occupies a total of 589,913 km^2^. The population is estimated to be 1,213,533 (2012). The main industries are fishing, timber, pulp and machinery (36–38). There are 22 prisons in the Arkhangelsk region (39).

In the Arkhangelsk region, there are 50 hospitals and 47 outpatient clinics (37). Officially there is one TB specialist (usually a doctor) in each hospital of the regions. All refer TB patients for treatment to the Arkhangelsk TB dispensary. District doctors send likely patients to the TB specialist, who provides the TB diagnostic process (fluorography, and three sputum tests). A situation in which there is no medical specialist is quite common. The dispensary is located in the town centre of Arkhangelsk and offers both inpatient and outpatient services. The outpatient departments provide treatment for TB patients without bacilli excretion. Patients with bacilli excretion, who are sensitive and carry resistant forms of *Mycobacterium tuberculosis*, are provided treatment in the inpatient departments. There is a separate department for patients with extensively resistant forms. Several social workers and psychologists provide support for patients during the entire treatment process. The Arkhangelsk region covers hundreds of square kilometres with the distance to Arkhangelsk varying from 30 to more than 600 km from different district centres. Access to medical help differs depending on remoteness and lack of infrastructure.

The alcohol consumption increases from south to north of Russia (the share of alcohol mortality in all mortality is 7% in Dagestan, 15% in the Murmansk region and Komi Republic and 23% in the Arkhangelsk region) (40).

### Participants

We used purposive sampling, and 32 informants (patients) (20 men and 12 women) aged 23–78 were included in the study. TB dispensary doctors selected the patients using the following inclusion criteria: patient with a history of DD and cough for more than 2 weeks. We used TB disease in the anamnesis as the exclusion criterion, for those patients who are ‘automatically’ suspected to have TB disease. We asked 35 patients initially, and three (one woman and two men) refused to participate citing lack of time. The patients had no experience of having TB before and had lived in the Arkhangelsk region for several decades. Ten patients (both men and women) had previously been in prison, 21 informants were alcohol dependent and 22 were smokers (according to medical documentation and self-report). We used the term ‘alcohol dependent’ for those who had physical and psychological dependence and ‘alcohol addict’ for those who had only psychological dependence according to ICD 10 (41). One person had no school education, 11 had secondary education and the remaining had higher education. Only 13 patients had a family; all others lived alone.

Eleven informants (medical doctors), all women aged from 35 to 53, were selected for the FGDs. Male doctors are very rare in the district. Inclusion criterion was that these doctors worked in different Arkhangelsk districts. They were selected randomly with no respect to their qualifications. All doctors agreed to participate in the FGDs.

### Data collection and analysis

Purposive and theoretical sampling were used to clarify and develop explored concepts and saturate them according to emerging codes and categories (33, 34). We asked questions during the data gathering and analysis that allowed us to clarify uncertainties, test our interpretations and build the emerging theory (33). Data collection and analysis were performed simultaneously. We conducted an initial analysis of each interview before performing the next one and in cases of upcoming important/interesting issues, we continued to explore/probe them in the next interview (33). When the flow of new information ceased and we could foresee the findings of the next interview, the process of data collection was considered saturated. We conducted in-depth interviews using a thematic interview guide. The interviews were focused on patients’ perceptions, knowledge and health-seeking behaviour related to TB and its management. In-depth interviews were used because of many existing sensitive and personal topics such as alcohol consumption and family relations. All interviews lasted 40–70 min and were audiotaped and transcribed verbatim by the PI. We conducted all interviews in the environment of the TB department, in a separate and quiet room. Patients and researchers used facemasks to prevent transmission of infection (demands of the hospital).

During the analysis several questions about the HSD appeared. For example, patients described incompetence of district doctors. We arranged two FGDs with district doctors to check the information and get more details about the situation from their point of view. These FGDs lasted 60–70 min each. Before initiating the FGDs, we evaluated the doctors’ knowledge in the TB field based on a simple test they were asked to complete about TB screening. The doctors knew how to manage TB-suspected patients (all showed excellent or good results). At the next step, we performed FGDs to understand doctors’ perceptions and experiences of the TB patients’ management field. We stopped data collection after 32 interviews and two FGDs. FGD data were analysed and coded in the same manner as the interviews.

We carried out data analysis on three levels: open, axial and selective coding (33). Open coding was used to scrutinise the data line-by-line to identify open codes. We labelled related codes and grouped them into categories. During the axial coding, we created categories by specifying the relationships between them. We identified two core categories that related to all other categories at the selective coding stage (33). We indicated in Table 1 how the codes emerge from citations to core categories.

**Table 1:** Table indicating the coding procedure from citations to core categories

Citation	Code	Subcategories	Category	Core category
*You freeze, you sweat, and the whole day is like that. Plus I did not sleep for 24 hours. I could have caught a cold. The cold is not a reason to seek medical help. Moreover, I had so much work that time!*	Symptoms of cold No awareness of danger Work as an obstacle for seeking medical help		These symptoms are not serious	Limited awareness of importance of contacting the health system
*Well, I thought I had caught a cold … fever and cough. I thought I would treat myself. Then it went further and further … it made me ask for help*.	Symptoms of cold Self-treatment Failure of self-treatment		Self-treatment	
*I had fever, 38*°*; I treated it by some pills, and I got relief and came down. Then fever became 39*°*, then 40*°*. I came to my daughter; she called for an ambulance*.	Fever as a symptom of cold Self-treatment Failure of self-treatment		Failure of self-treatment	
*They said that I have to go for fluorography, but I did not pay attention to it. I thought: later on, later on …. I have to work … the more I work the more money I will get*	Low attitude to own health Work as an obstacle for seeking medical help	Work/housing:	Factors influencing the health-seeking behaviour of patients	
*I have had a cough for more than one month. Well, such cough, like a normal one. I thought it was a cold …. not tuberculosis, but something else*.	Symptoms of cold Cough is normal thing Lack of knowledge about TB	Lack of knowledge about TB		
*I was in prison. I know these symptoms. I started coughing, but I thought that it was “a joke.”*	Prison as a special environment Informed about TB symptoms Low attitude to own health			
*I don't pay attention to my health, because I have to work from early morning till night. If I feel bad I drink to forget this*.	Low attitude to own health Work as an obstacle for seeking medical help Alcohol as a treatment	Low attitude to own health		
*When I drink, nothing is bad for me! Illness flies out with alcohol. You don't feel it. Alcohol softens everything, all diseases. When you drink you do not pay attention to illness. Well, today you sneeze, cough, but it will pass! In the morning you wake up, something squeaks, whistles; you groan but go anyway, then you forget about it during work*.	Alcohol as a treatment Alcohol as a life style	Alcohol use		
*This is the best prevention! (He showed the sign of drinking). Usually, I come from a shift, get drunk and it does not matter, if you have it or not [tuberculosis]*.	Alcohol as a treatment Alcohol as a life style			
*My wife cried that I should have gone to the doctor earlier. I was to blame that if I had gone to the doctor earlier our daughter would be OK. I'm guilty for the delay and I have infected my daughter*.	Wife blaming Child was infected Guilt for infection	Family support		
*I have got a daughter, 39 years. She told me: ‘Mama, go to the doctor, cure yourself!’ My mother said: ‘Go immediately, go to Arkhangelsk, get examined and cured’*.	Daughter's initiative to enter mama to hospital Fever is not a motivator			
*They support me, call very often and are interested in how I feel*.	Family support			
*I applied for the 2nd time. I lived in Moscow, so I came only in March, even though X-ray results were in the district in December. Doctors did not see it there, but in Arkhangelsk they did. I was sent to Arkhangelsk, when I was examined for the tenth time! They took sputum. I made fluorography in March and took pills for pneumonia till May*.	The second trial to get medical help Lived far from Arkhangelsk Doctor could discover disease Wrong diagnosis		Breach of diagnostic standards	Limited resources of the health system
*I visited a doctor three times! I had a fever 37,2*^*0*^ *, and headache. One doctor said: ‘Headache?’ ‘Yes!’ ‘Let it be’. ‘I can take sick leave for a month only, then my salary will be taken away’. He discharged me. Then I came again. There was another doctor. He sent me to a hospital, where I got injections. But the hospital was closed for reconstruction and I was discharged*.	Several attempts to contact the doctor Doctor's low attention to symptoms’ Doctor under strict administrative control Change of the doctor Infection in hospital		Treatment of wrong disease	
*They took sputum. I made fluorography in March and took pills for pneumonia till May*.	Failure of treatment		Failure of treatment	
*Our administration talks all the time about how we should complete treatment of all TB cases and how we should recognize new cases as soon as possible. But how can I do it, if they don't want to go to the doctor? A majority of patients are alcohol dependants. Very often we do not have enough doctors or nurses in the medical sites*.	Administration demands completed treatment Diagnosis in time is a criteria of effective work Alcohol is widespread among patients Lack of personnel		Factors influencing the health system delay	
*The patients are very hard, we have to visit their living places up to ten times to catch them and to convince them to take a test and treatment!*	Hard to find patients Hard to convince patient to take treatment	Knowledge of the health personnel		
*For sure, we prescribe two antibiotics if necessary. Patients don't want to be cured very often! How can we control them! It is hard to even catch them at home!*	Knowledge about right treatment Patients don't want to be treated Hard to find patients No tools to perceive patients			
*Our doctor is absent very often. Then we go to another one. But she might not know my situation and prescribes something at random. So, I don't like to go to other doctors and instead I try to wait for my doctor*.	Lack of doctors Doctors changed Wish to contact one doctor	Organisational problems		
*I came to the doctor, but did not enter the doctor's room because of a queue. There were 10 persons before me in the end of working hours*.	Long queue in policlinic Failed attempt to contact doctor			
*Fluorography works till 4 p.m. How shall a working person be able to do it?*	X-ray working time is inconvenient Patient have to work			
*Our work is not easy at all. Not everybody can work here. Much experience is necessary to work effectively. The system does not work by itself*.	Hard work in policlinic Special work People are the main factor in the policlinic	Experience of personnel		
*There are different patients in a medical district. Some make fluorography test regularly; others don't come for several years. There are alcohol dependants; they do not care at all*.	Patients are different Some patients don't follow prescriptions Alcohol-dependent patients are a problem			

Assigning of codes to the data was done by the PI and later shared and discussed with the other authors. The PI (V.K.) made the first coding in Russian, and then the codes were translated into English. All codes were discussed with E.J. in Sweden where we interpreted and recoded the data and developed properties and dimensions (33) of the categories. Being TB and public health specialists, other authors participated in discussing categories and core categories as well as the main idea of the article. The writing of memos was done during and after data collection as well as during research team work. The memos were important in the analysis, sorting and summarising of major findings and also in helping to stimulate thoughts about any additional data. We used member check to determine whether our interpretations matched with the participants’ opinions.

### Ethical considerations

The Ethics Committee of Northern State Medical University (Arkhangelsk) approved the study. Each participant was informed of the purpose of the study and signed a consent form. Every informant could refuse to participate in the interviews at any time without negative consequences. Only two informants rejected participating in the interviews; one after being requested to sign the written consent, another refused to provide any information because he was concerned about the consequences.

## Results

**Fig. 1:**
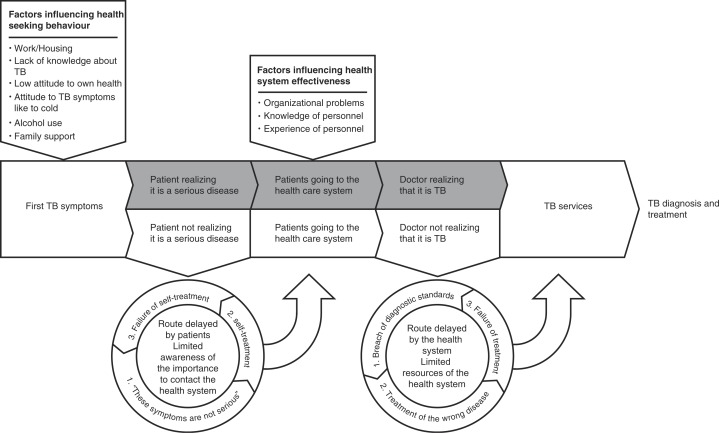
Model of the sickness trajectory, including categories influencing the process of TB diagnosis and treatment.

The study allowed us to create a substantive model and theory of DD among TB patients in the Arkhangelsk region. The theory indicates two core categories: first ‘limited awareness of the importance to contact the health system’, which could also be called ‘route delayed by patients’, and second ‘limited resources of the health system’, which could also be called ‘route delayed by the health system’. They reflect the perceptions of the participants (patients and doctors) concerning factors associated with DD of TB. A model ‘sickness trajectory in health-seeking behaviour among TB patients’ was developed to demonstrate the process of TB health-seeking behaviour including the two types of delay (Fig. 1). The categories could be seen as two vicious circles, which appear to be created during the health-seeking process, that is, on the way from onset of first symptoms to correct referral and correct treatment at the TB services. The aim of the trajectory is to describe the two types of PD and HSD; the interrelation between health-seeking behaviour of the patients, who sometimes do not think they have TB and doctors’ behaviour, who sometimes revealed they do not suspect TB among respiratory patients.

The two circles in Fig. 1 reflect the vicious circles of delay of correct TB diagnosis: the delay caused by the patients and the delay caused by the health system. Each route includes three subcategories describing the steps of making decisions (by patient or doctor, respectively, to the route). Factors that influenced PD and HSD are indicated in the rectangles of the figure. The shadowed arrow shows the ‘ideal route’ for patients without delay.

### Route delayed by patients: ‘limited awareness of the importance to contact the health system’

The first core category ‘limited awareness of the importance to contact the health system’ describes the individuals’ health-seeking behaviour. The route was an answer to the question ‘How do patients delay their visit to the health system?’ The route of PD included three main steps taken by the patients instead of contacting the health care (Fig. 1).

*Step 1:* Patients reacted to the first TB symptoms in similar ways as to a simple cold or disease (bronchitis, pneumonia, trauma, etc.), because TB does not have specific symptoms. Smokers considered cough as a symptom of smoking.

You freeze, you sweat, and the whole day is like that. Plus I did not sleep for 24 hours. I could have caught a cold. The cold is not a reason to seek medical help. Moreover, I had so much work that time! (Man, aged 52, alcohol dependent)

*Step 2:* The beginning of self-treatment, a typical behaviour for both genders. Patients used both traditional and modern ways (e.g. sauna and tablets) of trying to treat themselves. Waiting was a typical passive behaviour for those who are alcohol dependent. They believed that this disease would disappear by itself. Some patients experienced relief after self-treatment.

Well, I thought I had caught a cold … fever and cough. I thought I would treat myself. Then it went further and further … it made me ask for help. (Man, aged 36, alcohol addict)

*Step 3:* Failures of self-treatment or waiting with an inability to work or live a usual life. Very often they had to call emergency services. All participants were divided into three categories: 1) people who worried about the symptoms and came to the health system, 2) people who changed a treatment (such as from one drug to another) and 3) those who waited for increasing symptoms.

I had fever, 38°; I treated it by some pills, and I got relief and came down. Then fever became 39°, then 40°. I came to my daughter; she called for an ambulance. (Woman, aged 59, alcohol dependent)

#### Factors influencing the health-seeking behaviour of patients

These factors were answers to questions such as ‘Why do patients delay their visit to the health system?’

*Work/housing.* Participants perceived working or keeping their house as incompatible with seeking medical examination, because working hours of polyclinics (laboratories especially) were inconvenient. They had to earn money for their families and had no time for illness and examinations. Patients had to wait for their turn for 3–4 hours in the polyclinics and could not ask their boss for leave from work for such a long time. In addition, the bosses did not like to lose a worker for several days because of sick leave. Salary for sick leave was usually less than the salary for real work.

They said that I have to go for fluorography, but I did not pay attention to it. I thought: later on, later on … I have to work … the more I work the more money I will get (Man, aged 51, alcohol dependent).

*Lack of knowledge about TB*. People did not know that TB has the same symptoms as a common cold, and said, *It is not likely for me to have TB!* They looked for ‘serious’ symptoms, because ‘TB is a serious disease and therefore must have serious symptoms’. This led to a passive behaviour until such complications as hyperthermia or severe weakness appeared. Some people knew about TB symptoms and had experience with TB patients (friends, neighbours, other person in prison, etc.), but this knowledge did not always result in an early visit to a health centre.

I have had a cough for more than one month. Well, such cough, like a normal one. I thought it was a cold … not tuberculosis, but something else. (Woman, aged 45, alcohol dependent)

I was in prison. I know these symptoms. I started coughing, but I thought that it was “a joke”. (Man, aged 48, alcohol dependent, prison in past)

*Low attitude to own health.* People lost money because they were not working during the visit to the health centre, which was often situated in another town. Moreover, they had to pay for a hotel, tickets, and so on. People chose work or family before their health, which was not valued until a person got a serious disease, incompatible with normal life:

I don't pay attention to my health, because I have to work from early morning till night. If I feel bad I drink to forget this. (Man, aged 52, alcohol dependent)

*Alcohol use.* Almost all men and half of the women reported that they drank alcohol (multiple drinks) more often than once a week (according to interview data), had binge drinking and had alcohol dependence (according to medical records). Only one man did not do so, but he had a ‘rich alcohol experience’ in the past. Some used alcohol as treatment. These groups could be a source of aggregating and transmitting different infections including TB.

When I drink, nothing is bad for me! Illness flies out with alcohol. You don't feel it. Alcohol softens everything, all diseases. When you drink you do not pay attention to illness. Well, today you sneeze, cough, but it will pass! In the morning you wake up, something squeaks, whistles; you groan but go anyway, then you forget about it during work. (Man, aged 51, alcohol dependent).

This is the best prevention! (He showed the sign of drinking). Usually, I come from a shift, get drunk and it does not matter, if you have it or not [tuberculosis]. (Man, aged 53, alcohol dependent, jobless)

Alcohol dependence brought changes of the personality (i.e. decrease in self-criticism) (42) resulting in reduced social responsibility and delayed diagnosis. The changes of personality were impossible to find directly at the interviews with the patients, but this was indicated in the sense that their behaviour did not match their stories for alcohol-dependent persons (according to medical records). Informants stated that drunken persons never go to a doctor, but treat all hangovers by taking more alcohol and thus a vicious circle was formed. Persons who had no alcohol dependence reported HSD but not PD.

*Family support.* The family support usually prevented DD. Participants worried about family members and sought medical care to protect the children. Very often they blamed themselves for being a source of infection. Some people were afraid to infect people around them so that motivated them to apply for medical care. There was no obvious social stigma, but self-accusation motivated some to early TB diagnosis.

My wife cried, that I should have gone to the doctor earlier. I was to blame that if I had gone to the doctor earlier our daughter would be OK. I'm guilty for the delay and I have infected my daughter. (Man, aged 38, alcohol addict)

Women sent the family member to the doctor. Perhaps women were culturally responsible for supporting the well-being of the family members, while men aimed to earn money to feed their family. Almost all men reported PD, but a majority of the women reported HSD. The family never rejected the patient after the diagnosis of TB, but provided support, maintained the relation and tried to help to manage the disease.

I have got a daughter, 39 years. She told me: ‘Mama, go to the doctor, cure yourself!’ My mother said: ‘Go immediately, go to Arkhangelsk, get examined and cured’. (Woman, aged 59, alcohol dependent, jobless).

They support me, call very often and are interested in how I feel. (Woman, aged 26, alcohol addict, student)

The factors looked like explanations of weak motivation rather than factors that determined the patients’ behaviour. The participants did not seem to regret the delay, but rather looked for a reason for delay because there are contradicting points: from the one side, they have no time for contacting the health system caused by necessity for work or housekeeping; from the other side, they described binge drinking for several days that interrupted their daily life.

### Route delayed by health system: ‘limited resources of the health system’

The second core category ‘limited resources of the health system’ was an answer to the question ‘How does the general HSD the referral of the patients to the TB system?’ This core category is described with a circle, and illustrates when the patient was referred the wrong way or was treated for the wrong disease. This circle consists of the three following steps: 1) breach of diagnostic standards, 2) treatment of wrong disease and 3) failure of treatment (Fig. 1).

*Step 1:* Some doctors breached the diagnostic standards, for example, sputum examination might not follow the standard.

*Step 2:* As a result, TB was not discovered and a wrong diagnosis was stated; the wrong disease was treated. This process could last for 1 month to more than 6 months. The patients might get relief for some time, but then their condition worsened. Breach of diagnostic standards is hardly conscience; it is rather a result of a sum of actions of different factors such as doctor's absence, a lack of X-ray results, doctor's overload, and so on. In addition, patients could discontinue treatment at any time.

*Step 3:* Treatment failure often followed incorrect treatment/wrong diagnosis. Patients could change their doctor because of lack of trust or because of the doctor's absence (vacation, sick leave, etc.). At this stage, a new doctor establishes a new diagnosis, which could be right or wrong. A correct decision sent the patient to the TB dispensary, while a wrong decision caused the vicious circle to be repeated again and again.

I applied for the 2nd time. I lived in Moscow, so I came only in March, even though X-ray results were in the district in December. Doctors did not see it there, but in Arkhangelsk they did. I was sent to Arkhangelsk, when I was examined for the tenth time! They took sputum. I made fluorography in March and took pills for pneumonia till May. (Woman, aged 26, alcohol addict, student)

I visited a doctor three times! I had a fever 37.2°, and headache. One doctor said: ‘Headache?’ ‘Yes!’ ‘Let it be’. ‘I can take sick leave for a month only, then my salary will be taken away’. He discharged me. Then I came again. There was another doctor. He sent me to a hospital, where I got injections. But the hospital was closed for reconstruction and I was discharged. (Woman, aged 48)

#### Factors influencing the HSD

This category gave an answer to the question: ‘Why does the HSD the referral of the patients to the TB system?’ These factors were organisational problems, lack of knowledge and experience. Doctors described the health system as lacking resources and not being well organised. They blamed the administration for organisational problems, for example wrong working hours of the X-ray departments. The administration required the doctors’ full personal responsibility for each patient's diagnosis and treatment. Moreover, doctors blamed patients for alcohol consumption and moving from one district to another without any notification.

Our administration talks all the time about how we should complete treatment of all TB cases and how we should detect new cases as soon as possible. But how can I do it, if they don't want to go to the doctor? A majority of patients are alcohol dependent. Very often we do not have enough doctors or nurses in the medical sites. (Woman, doctor, aged 48)

The patients are very hard [to find], we have to visit their living places up to ten times to catch them and to convince them to take a test and treatment! (Woman, doctor, aged 52)

*Knowledge of the health personnel.* According to the standard TB diagnostic process, the doctor should prescribe a sputum microscopy and a chest X-ray if a patient has a cough for more than 2 weeks. If the result of microscopy is negative and the X-ray picture is pneumonia-like, the doctor should prescribe a course with two antibiotics (semisynthetic penicillin +macrolides) with subsequent X-ray examination. The improvement seen in X-rays after antibiotic therapy is considered a sign of TB absence. There are no strict standards for alcohol dependence in general practice. Persons suspected of alcohol dependence could only receive recommendations to contact psychiatric services according to Russian law.

The doctors discussed patients’ willingness to take the antibiotics. They described patients who did not agree to take treatment as they lived in the street, ignoring local customs or official rules, and did not show any social interest. Patients did not mind taking pills, but they did not want to follow any regime. Sometimes, they refused treatment after improvement or receiving side effects of the antibiotics.

For sure, we prescribe two antibiotics if necessary, but patients are not interested in taking the treatment. Patients don't want to be cured very often! How can we control them? It is hard to even find them at home! (Woman, doctor, aged 38)

*Organisational problems.* There was a lack of staff in some remote districts. If a doctor was ill or on vacation, other doctors had to care for patients from two districts. Patients found themselves in long queues, or it was impossible to get the doctors’ attention, leaving the patients at a loss. Moreover, some patients did not contact another doctor, but were waiting for their district doctor to come back and this then delayed the diagnosis. This led to a decrease of patients’ trust in the health system and as a result lowered their motivation to seek medical help, which illustrates the interrelation of PD and HSD.

Our doctor is absent very often. Then we go to another one. But she might not know my situation and prescribes something at random. So, I don't like to go to other doctors and instead I try to wait for my doctor. (Woman, aged 52, alcohol addict)

I came to the doctor, but did not enter the doctor's room because of a queue. There were 10 persons before me at the end of working hours. (Woman, aged 39, alcohol addict)

Working hours of the X-ray examination departments coincide with working hours of the majority of people. Sometimes the necessity to be present at work hindered a visit for examination.

Fluorography works till 4 p.m. How shall a working person be able to do it? (Woman, doctor, aged 37)

TB suspicious patients should be sent to TB services. A patient with negative sputum might be sent back with a notification that two courses of antibiotics should still be provided. Some doctors reported that even the TB dispensary might disobey these requirements if the symptoms are obvious.

The organisational problems seemed to be crucial. This is the point where the second vicious cycle starts. It is easy for patients to end up in the vicious circle instead of meeting a doctor, who could diagnose and start treatment. The first meeting with the doctor in the health centre and the second meeting with the doctor at the TB services seem to be crucial for being diagnosed and treated.

*Experience of personnel.* Doctors described that their work demanded special communication skills, such as communication with patients who have such complex problems as alcohol dependence. The diagnostic process was complex and a patient should follow the treatment path. Some patients followed the prescribed pathway, but others could discontinue treatment at any time because of alcohol use and no place to live, and so on.

Our work is not easy at all. Not everybody can work here. Much experience is necessary to work effectively. The system does not work by itself. (Woman, doctor, aged 48)

There are different patients in a medical district. Some make fluorography test regularly; others don't come for several years. They are alcohol dependent; they do not care at all. (Woman, doctor, aged 44)

The administration demanded control of every patient in terms of progress of treatment. The doctors had to report a definite result for each case. Doctors did not have any resources to influence patients except for the use of personal communication skills. They did not offer any suggestions on how to resolve the situation but just described it.

Some of the blame placed on the patients, such as not being at home, interrupting treatment early, etc. could be seen as obstacles to the doctors achieving their professional targets. The doctors were thoroughly controlled by the administration, there were often few of them and they frequently filled in for each other. PD and HSD interrelation could also be caused by low patients’ trust in the health system.

## Discussion

The first core category ‘limited awareness of the importance to contact the health system’ together with the category ‘factor influencing the health-seeking behaviour’ gave answers to the questions: ‘Why and how patients delayed their visit to the health system?’ Patients showed a rather low awareness of the importance of contacting the health system, possibly also indicating low trust in the health system. Patients seemed to look for an acceptable reason or explanations for not going to the health system, when symptoms of TB first appeared. This behaviour may be based on a passive position of the person, overpowered by hopelessness (43). This sometimes led to patients entering a vicious circle, where they tried to treat themselves, instead of contacting the health services directly.

The second core category ‘limited resources of the health system’ describes factors influencing the DD, caused by the health system and how the route was delayed by the system itself. It gave answers to the questions: ‘Why and how do the HSD the referral of patients to the TB system?’ We have described such factors as organisational problems, knowledge and experience of personnel. The system was described as having a lack of resources and as being poorly organised and integrated. Doctors blamed administration and patients for the situation. The rather negative attitude of the doctors appears to stigmatise alcohol-dependent patients, which might lead to breach of relationship and trust between doctor and patient.

The HSD was manifested in the vicious circle, named ‘route delayed by the health system’ consisting of such steps as ‘breach of diagnostic standards’, ‘treatment of a wrong disease’, and ‘failure of treatment’. Patients perceived HSD as being caused by doctors’ incompetence. According to our data, doctors showed quite good competence. HSD is not huge in the Arkhangelsk region: the HSD median time in passive case findings was less then 2 weeks, while PD was 6 weeks in 2005–2011 (23). It is possibly that HSD are caused by the sum of different factors in the health system, such as changing of doctors, absence of X-ray results, minimal doctor's attention caused by overload or by other factors. Negative PD and HSD interrelation was possibly influenced by low patients’ trust for the health system.

We have found similar types of patient DD, including social and economic factors as well as alcohol dependence, as other researchers before us (1, 4, 11, 14, 15, 18). The delay was often related to low awareness and knowledge of TB as also earlier reported (4, 5, 8, 9). Fear of being infected leads to isolation and stigmatisation of TB patients (16), and stigma plays a negative role in health care seeking (10). Surprisingly, we did not find that stigma played any significant role for delaying TB diagnosis in our region. Moreover, some patients who suspected that they were infected with TB were concerned of the potential to infect others. This motivated some of them to contact the health system early. People who belonged to a less vulnerable group described concerns about future contacts and communication with workmates or neighbours. However, they also reported that isolation did not last for long. Such a situation is typical for a village with a small and closed society. TB was common for special groups, such as poor and homeless people, alcohol addicts, etc. (4). We did not find any cases where the family (or other relatives) rejected a family member who had been diagnosed with TB. It seemed to be atypical for this society. Although alcohol dependence and addiction is rather common in the Arkhangelsk region, it was difficult to see any direct stigmatisation of these types of patients by the doctors. They rather complained about the difficulties to reach these patients.

Women were not reported more vulnerable in our setting compared to reports from settings in other countries (3, 44–46). In addition, they reported less PD. We discovered that alcohol dependence among women made them more vulnerable and those women who abused alcohol are less likely to approach health services. This agreed with other data (42). Screening for alcohol dependency in women in the general health system might prevent TB PD for them.

We found similar influencing factors for PD and HSD in our study similar to other researchers, who described longer DD in health centres compared to lung clinics. The reasons for this were said to be breach of diagnostic standards (6, 18, 47); that some TB cases were underreported because of negative results of microscopy, and poor dialog between different levels of the health system (4, 5, 8, 9).

Some researchers consider the lack of new diagnostic tests in clinics a risk factor for TB DD (7), but we found that the TB system has enough such tests (LPA, MGIT and GeneXpert) in the Arkhangelsk region. So, we cannot associate DD in our setting with lack of diagnostic tests. In the Arkhangelsk region such factors as patients’ perceptions, knowledge, trust, and health-seeking behaviour related strongly to TB DD.

We used theoretical sampling for data gathering, based on facts of DD. The majority of our informants came from vulnerable groups of people [similar to information from other researchers (11, 12, 18–21)] and also had alcohol problems similar to the findings of Oeltmann et al. (22).

The strengths of this research include the multi-faceted research team that provided a good level of validity and allowed data triangulation. Moreover, we used the FGDs with district doctors to answer questions raised in the in-depth interviews, and we acquired two-sided perceptions of the DD process: from patients and doctors. We used theoretical sampling to gather data and included different patients without selection by age, education or social level, etc. For instance, we did not especially select persons with alcohol problems; they were just typical for TB patients. In-depth interviews allowed us to reach a deeper level of trust, and also consequently information about such sensitive topics as alcohol use and family relations.

Limitations included the need to wear facemasks, which reduced face-to-face communication but was a necessity for work in the TB department. This is the routine for a new patient during 1–2 weeks after receiving the diagnosis of TB, because of the infection regime. We performed FGDs with doctors only, not with nurses or doctor assistants (‘feldshers’). Nurses and doctor assistants follow doctors’ directives in our context. If nurses or feldshers work independently (in case of doctor's absence), they take all responsibility for the work as a doctor. There is no team decision. Another limitation is that one person conducted, transcribed and coded the interviews initially.

## Conclusion

We discovered two core categories that emerged from data and described two vicious circles of DD routes among patients with TB in the Arkhangelsk region, namely ‘limited awareness of the importance to contact the health system’ and ‘limited resources of the health system’. The model ‘sickness trajectory in health-seeking behaviour among TB patients’ shows the two core categories and other categories and their interrelations. The model shows two vicious circles of PD and HSD, based on our findings (categories). The circles seem at first to be separate processes, but the purpose of the health-seeking behaviour is to be diagnosed and treated. The PD might be interpreted as the populations’ low awareness of the importance of contacting the health system. This low awareness could be explained as either limited awareness or knowledge about TB disease among the population, but it could also be seen as low trust in the health system, leading to avoidance or late seeking of care. The HSD also seems to have its main weakness in the health centres, where cases are not properly diagnosed and referred to a TB specialist. The whole organisation of the system seems too rigid and needs thorough reform. Men reported more frequently patients’ delay, whereas women more often reported experiencing HSD. These findings will allow the health system to concentrate efforts on involving people in early medical examination and on increasing alertness to TB among patients. Also, improving the health system performance in the districts can save resources and decrease costs, making these efforts both effective and beneficial.

**Vladimir N. Kuznetsov** is a PhD candidate in the Institute for Health and Society, University of Oslo, Oslo, Norway and associate professor of department of psychiatry of Northern State Medical University, Arkhangelsk, and International School of Public Health, Arkhangelsk, Russia.

**Andrej M. Grjibovski** is a professor at the Department of International Public Health, Norwegian Institute of Public Health, Oslo, Norway and director of International School of Public Health, Arkhangelsk, Russia.

**Andrej O. Mariandyshev** is a professor at the Department of Tuberculosis, Northern State Medical University, Arkhangelsk, Russia.

**Eva Johansson** is a professor at the Karolinska Institute, Stockholm, and Nordic School of Public Health, Gothenburg, Sweden.

**Gunnar A. Bjune** is a professor at the Institute for Health and Society, University of Oslo, Oslo, Norway.
